# Understanding nanostructure formation from first principles

**DOI:** 10.1186/1758-2946-4-S1-O13

**Published:** 2012-05-01

**Authors:** Eva Rauls

**Affiliations:** 1 Universität Paderborn - Theoretische Physik, N3.315, Pohlweg 55, 33098 Paderborn, Germany

## 

In recent years, nanotechnology found its way into various fields of our life, ranging from applications in pharmacy or food technology to miniaturized electronic or optical devices.

Functional surface coatings are among the most well known applications.

Bottom up design is one of the most promising possibilities to build new materials with specifically tailored properties. During the last decade, intense research has been carried out in this area, and many applications have already been put into practice. However, in the world of nanostructures, there are still many effects just waiting to be explored and utilized for new applications.

In computational materials science, we approach the field from the theoretical side in order to gain a deeper and fundamental understanding of structure formation and how the involved processes can efficiently be improved.

In my talk, I will give an overview of some of our recent projects in this field. In the first part, I will discuss the self-organisation driven formation of a covalently bonded molecular network and the catalytic role the surface plays for the reaction between two organic molecules. Imidization reactions - well known in chemistry and biology for a long time, but rather newly discovered by surface scientists - constitute an outstanding tool for the design of nanostructures.

In the second part of my talk, I will show some first results we obtained for the physical properties of metallo-porphyrines adsorbed on a Au-surface and conclude with a brief overview of some of our other fields of research.

